# An ethnoveterinary study on medicinal plants used by the Buyi people in Southwest Guizhou, China

**DOI:** 10.1186/s13002-020-00396-y

**Published:** 2020-08-17

**Authors:** Yong Xiong, Chunlin Long

**Affiliations:** 1 College of Life and Environmental Sciences, Minzu University of China, Beijing, 100081 China; 2 Key Laboratory of Ethnomedicine, Ministry of Education of China, Minzu University of China, Beijing, 100081 China; 3 School of Ethnomedicine & Ethnopharmacy, Yunnan Minzu University, Kunming, 650500 China; 4 Kunming Institute of Botany, Chinese Academy of Sciences, Kunming, 650201 China

**Keywords:** Ethnobotany, Buyi people, Ethnoveterinary medicine, Qianxinan Prefecture

## Abstract

**Background:**

The Buyi (Bouyei) people in Qianxinan Buyi and Miao Autonomous Prefecture, Southwest Guizhou, China, have used medicinal plants and traditional remedies for ethnoveterinary practices, such as treating domestic animals during livestock breeding, since ancient times. However, the unique ethnoveterinary practices of the Buyi have rarely been recorded. This study aimed to identify the plants used in their traditional ethnoveterinary practices, and to propose suggestions for future conservation and sustainable use of this knowledge.

**Methods:**

Ethnobotanical fieldwork was conducted in 19 villages/townships in Qianxinan Prefecture between 2017 and 2018. Data were collected from the local Buyi people through semi-structured interviews and participatory observations. The informant consensus factor (FIC) and use reports (URs) were utilized to evaluate the consent of the current ethnoveterinary practices among the local communities, and 83 informants were interviewed during the field investigations. Plant samples and voucher specimens were collected for taxonomic identification.

**Results:**

A total of 122 plant species, belonging to 60 families and 114 genera, were recorded as being used in ethnoveterinary practices by the Buyi people. The most used ethnoveterinary medicinal plant (EMP) parts included the roots, whole plant, and bulb, and the most common preparation methods included decoction, crushing, and boiling. Some EMPs, such as *Quisqualis indica* and *Paris polyphylla*, have special preparation methods. The informant consensus factor (FIC) and use reports (URs) of the EMP species were analyzed. Twenty EMP species with the highest URs were noted as having particular importance in the daily lives of Buyi people in Qianxinan Prefecture.

**Conclusion:**

In this study, we identified traditional ethnoveterinary knowledge of the medicinal plants among the Buyi communities in Qianxinan Prefecture. This knowledge has previously been limited to local vets, herders, and aged community members. Plants with important medicinal uses need to be validated phytochemically and pharmacologically in the future, to develop new alternative drugs for veterinary purposes.

## Introduction

Ethnoveterinary medicines are generally defined as being used for folk skills, beliefs, knowledge, practices, methods related to animals’ health, and to cure various ailments in the ethnic group areas [1]. Ethnoveterinary medicine is the overall scientific term for traditional animal health care, which provides low-cost alternatives to allopathic drugs. The utilization of traditional ethnoveterinary remedies provides a cheaper, easier, and more sustainable alternative to synthetic drugs and pharmaceuticals [2]. In China, traditional knowledge of ethnoveterinary medicine originates from indigenous peoples’ daily livestock management and the long history of these practices, such as those recorded in *A Complete Collection of Veterinary Herbal Medicines* [3], *Chinese Veterinary Medicine* [4], *Tibetan Veterinary Drugs and Instrument Atlas in Zuoergai Plateau* [5], and *Mongolian Veterinary Research* [6]. Ethnoveterinary medicine has been used for a long time, especially in countries with more developed animal husbandry, such as Italy [7], Spain [8], Navarra [9], East Africa [10], Pakistan [11], Brazil [12], India [13], and China [14, 15]. Traditional and low-cost methods to treat animal diseases instead of synthetic drugs are often desired.

According to the 2010 census in China, the Buyi (also named Bouyei) ethnic group was the 11th largest of the 55 ethnic groups recorded, with a population of approximately 2.87 million [16]. The Buyi people are mainly distributed in the southwest of China, including Guizhou, Yunnan, and Sichuan provinces. More than 98% of the total population of Buyi people reside in Qiannan and Qianxinan prefectures of Guizhou Province. The remaining Buyi people are scattered in Luoping and Maguan counties of Yunnan, Ningnan County of Sichuan, and a small number in Vietnam. Qianxinan Buyi and Miao Autonomous Prefecture is the main dwelling places of the Buyi people. Qianxinan Prefecture comprises two cities (Xingyi and Xingren), six counties (Ceheng, Wangmo, Anlong, Pu’an, Zhenfeng, and Qinglong), and one new district (Yilong) since 2018 (http://xzqh.mca.gov.cn/map), and is rich in indigenous Buyi culture and resources. For example, Ceheng County is the “First County of Buyi people in China,” and Wangmo County is the most distinctive region of the Buyi people. Anlong, Zhenfeng, and other counties are also rich in Buyi traditional culture and knowledge. Buyi People’s Museum and Buyi People’s Textile Museum are located in this region.

The Buyi is one of the ethnic groups in southwest China. They primarily subscribe to polytheistic animism, believing that their ancestors, spirits, and ghosts are able to influence the health of people, the success of their clan, the bounty of harvest, the productivity of cattle, and the harmony of community. They gradually formed their epistemologies, such as the value of harmonious relationship with nature during a long process of production and practice. In the belief-system of Buyi people, they usually have pantheistical adorations, such as habitats (e.g., sacred mountains), plants (e.g., divine arbors, bamboo), animals or mythological creatures (e.g., fish, dragon), and natural elements (e.g., fire). Sacred trees occupy a very important position in the traditional culture of Buyi people. Various arbor species may be endowed with supernatural power and treated as sacred trees, including *Ficus* spp., *Celtis biondii*, *Ulmus parviflora*, and *Aesculus chinensis* [17]. In Ponai Village, Ceheng County, for example, *Aesculus chinensis* is a village protected tree species, and there are many red damask slices hanged on the trees. People pray here for good weather and safety of the village. The villagers can collect its seeds as a medicine to treat diarrhea of piglets. All of these ideas played a positive role in environmental protection and sustained the Buyi ethnic culture over time. Most of the Buyi villages are located in an area with a well-preserved natural habitat, good ecological environment, and rich biodiversity, which may provide a favorable foundation for medicinal plant resources for the local folk doctors. They have a long history of using medicinal plants for animals as part of their indigenous medical system. The villagers depend on natural habitat for gathering and collecting products for household consumption such as vegetables and medicinal herbs. The villagers also generate some income from the sale of timber, fuel wood, and non-timber products. Local people’s main agricultural income is derived from selling rice and maize.

Qianxinan Prefecture has a southern subtropical climate. This complex topography and diversity of climates significantly contributes to the richness of local biodiversity. The Buyi people are a rice-farming ethnic group, living mainly in the areas near forests and rivers [18]. A number of paddy fields are located in this region. Livestock and crop cultivation have a close linkage and interaction in the Buyi agricultural system. In order to provide protein supplies for themselves and as an economic resource, each family commonly raises chicken and pigs. Residents also keep other species of animal, such as dogs and cats, to guard their houses. In the field investigation, although the use of domestic animals as labor has been decreasing with the mechanization of agriculture and the increase in transportation facilities, the use of domestic animals as food and economic resources are increasing in Buyi villages.

The domestic animal species are pig (*Susscrofa domestica*), cattle (*Bos taurus domestica*), buffalo (*Bubalus arnee*), goat (*Capra hircas*), sheep (*Ovis aries*), donkey (*Equus asinus*), horse (*Equns caballus*), chicken (*Gallus domesticus*), duck (*Anas platyrhynchus* var. *domestica*), and goose (*Anser cygnoides domestica*). According to the economic survey report of the Qianxinan Prefecture, there were 1.222 million pigs, 0.483 million cattle, 0.49 million goat/sheep, and 8.49 million poultry in the prefecture (http://www.qxn.gov.cn/zfxxgk/fdzdgknr/ghjh/zfgzbg/). The Buyi people have mastered the use of traditional medicinal plants for the treatment of livestock diseases. Although local residents do not usually have the chance to use ethnoveterinary remedies to treat their livestock, experienced veterinarians still retain the traditional knowledge and practices.

However, there have been no studies on the medicinal plants for ethnoveterinary purposes used by the Buyi people in Qianxinan Prefecture. With the social and economic development, modern agricultural machinery has replaced human and animal labor in many rural areas. The younger generations leave rural counties for both education and work, and they are less willing to inherit traditional professions and learn traditional knowledge from their parents. Traditional ethnoveterinary knowledge is mainly transferred orally between generations, thereby being at a high risk of extinction. It will also disappear with the death of experienced older generations. Thus, traditional knowledge will be lost forever. This kind of extinction is irreversible. It is, therefore, urgent to carry out the investigation and research work of ethnoveterinary medicine. Consequently, this study aimed to collect and document the traditional ethnoveterinary knowledge and practices in the Buyi area.

## Materials and methods

### Study area

**Fig. 1:**
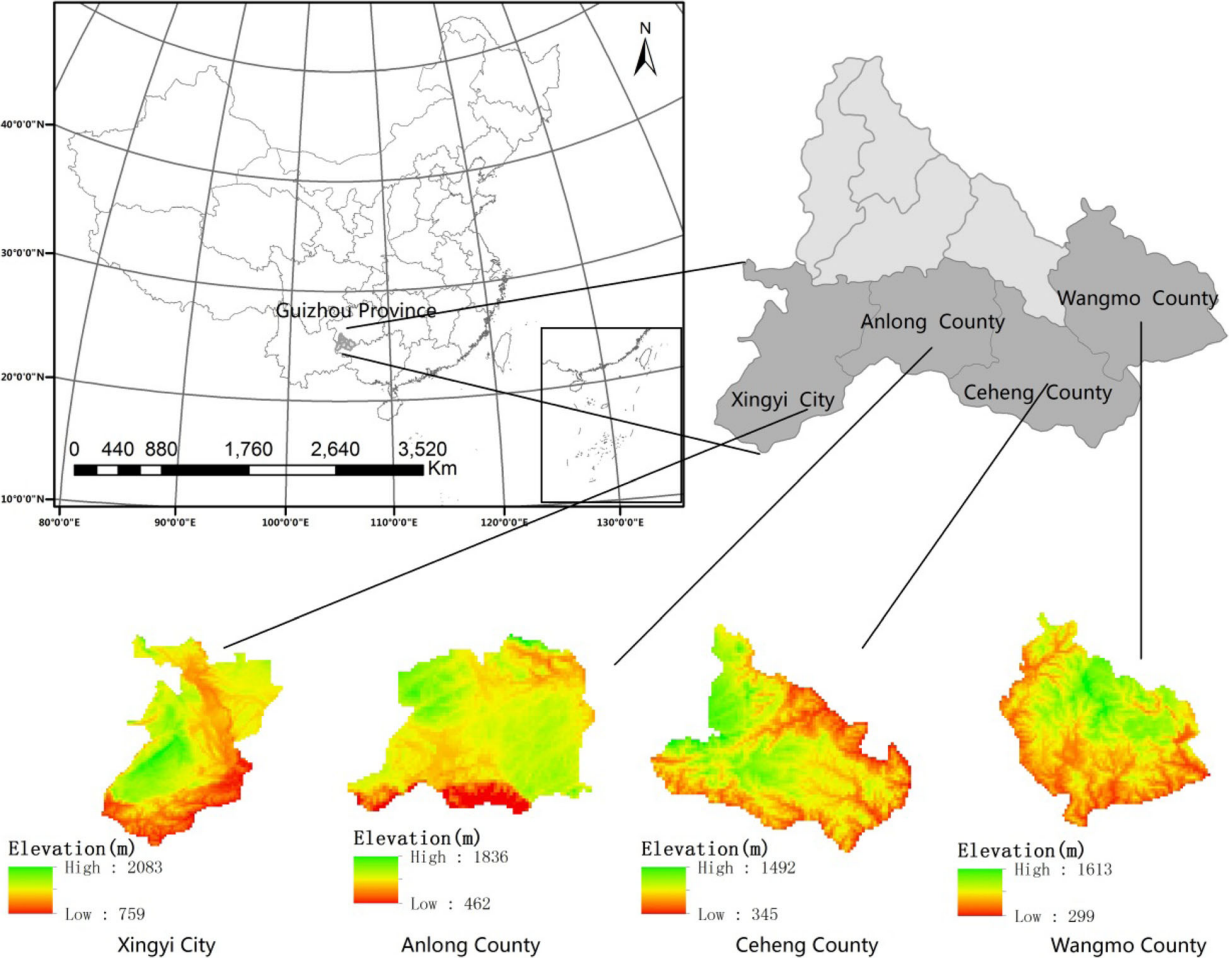
Map of the study area and location in Qianxinan Prefecture

The current study was undertaken in Qianxinan Buyi and Miao Autonomous Prefecture, Guizhou Province, China (Fig. 1). The prefecture is situated in the southwest end of Guizhou Province, between 104° 35′~106° 32′ E and 24° 38′~26° 11′ N. The total area of Qianxinan Buyi and Miao Autonomous Prefecture (capital in Xingyi City) is approximately 16,804 km^2^. The terrain of this Autonomous Prefecture is high in the west and low in the east. The highest point on the Pengzha plateau peak of Xingyi City is 2207.2 m above sea level. The lowest point is the Daluo estuary of the Hongshui River in Wangmo County, with an elevation of 1000~2000 m [19]. The Autonomous Prefecture has large topographic relief and complex landforms, and it can be divided into five different landform areas: low mountain erosion mountain canyon areas, Karst plateau grooved dam areas, Karst erosion plateau, Karst erosion mountain area, and erosion of mountain valley areas. The typical climate is humid subtropical monsoon, the annual average temperature is 13.8~19.4 °C, and the prefecture has three large rivers, Nanpan, Beipan, and Hongshui Rivers.

The survey area included 19 villages, 4 local herbal medicinal markets, 3 traditional animal breeding farms, and 2 traditional museums from Xingyi City, Anlong County, Cehong County, and Wangmo County (Table 1).

**Table 1:** Investigation sites in Qianxinan Buyi and Miao Autonomous Prefecture

County/City name	Village and township	Local herbal medicinal market	Traditional museum	Traditional breeding farm
Anlong County	A’neng Village, Tongsa Village, Guantun Village, Longguang Township.	Anlong County market	Anlong County Museum	Honglong breeding farm
Cehong County	Ceyang Village, Chayuan Village, Banwan Village, Ponai Township.	Ceheng County market	Zhonghua Buyi People Museum	Rongduzhen Yangbo Yuan
Wangmo County	Sanglang Village, Nanbei Village, Luwang Village, Mashan Village, Daguan township, Liudong Township.	Wangmo County market		Sanglang Rural Cooperative Pig Farm
Xingyi City	Huangcao Township	Panjiang East Road market.		

**Fig. 2:**
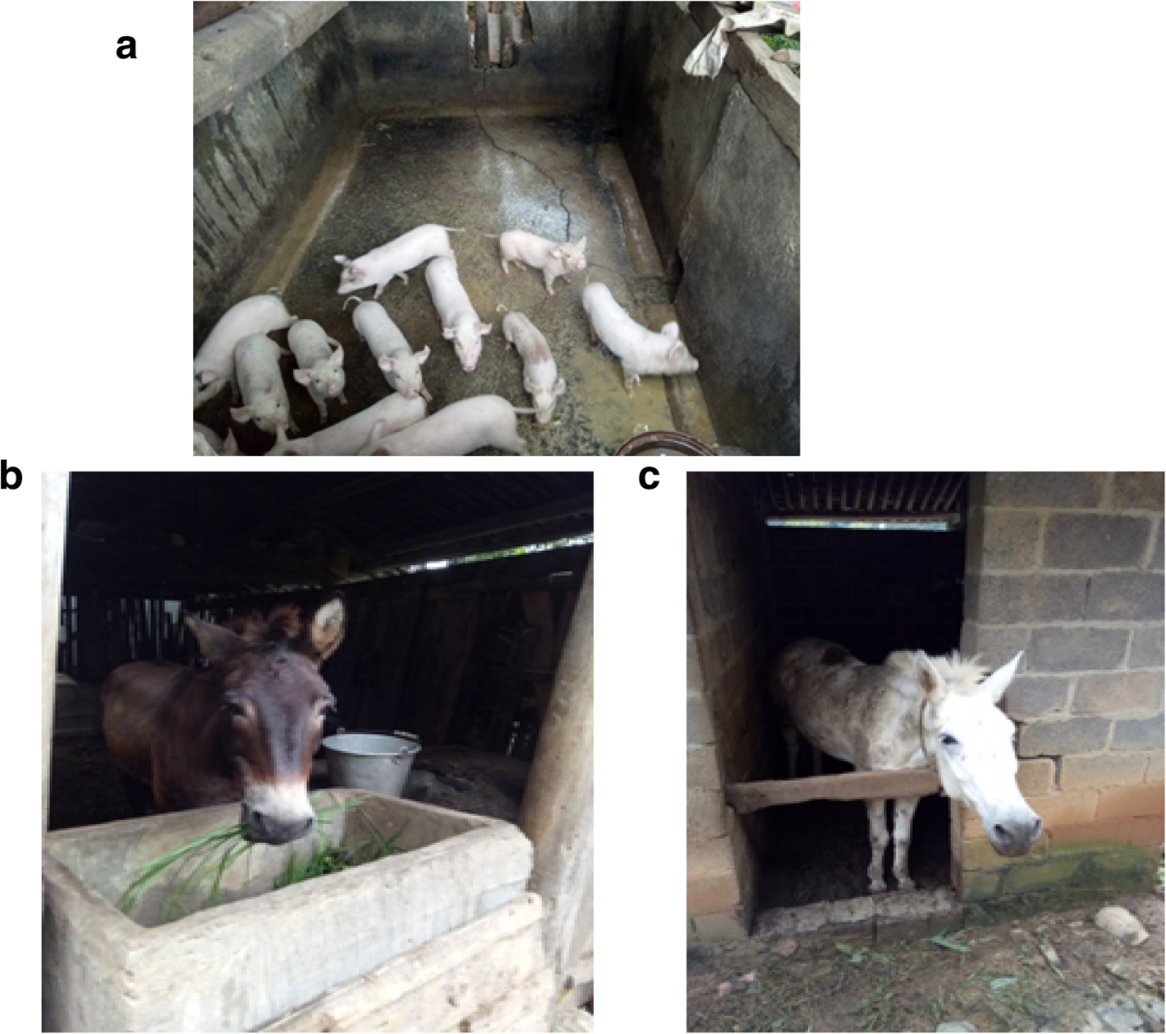
Local domestic animals raised by the Buyi people in Sanglang Village. **a** Piglet duroc. **b**, **c** Mule

**Fig. 3:**
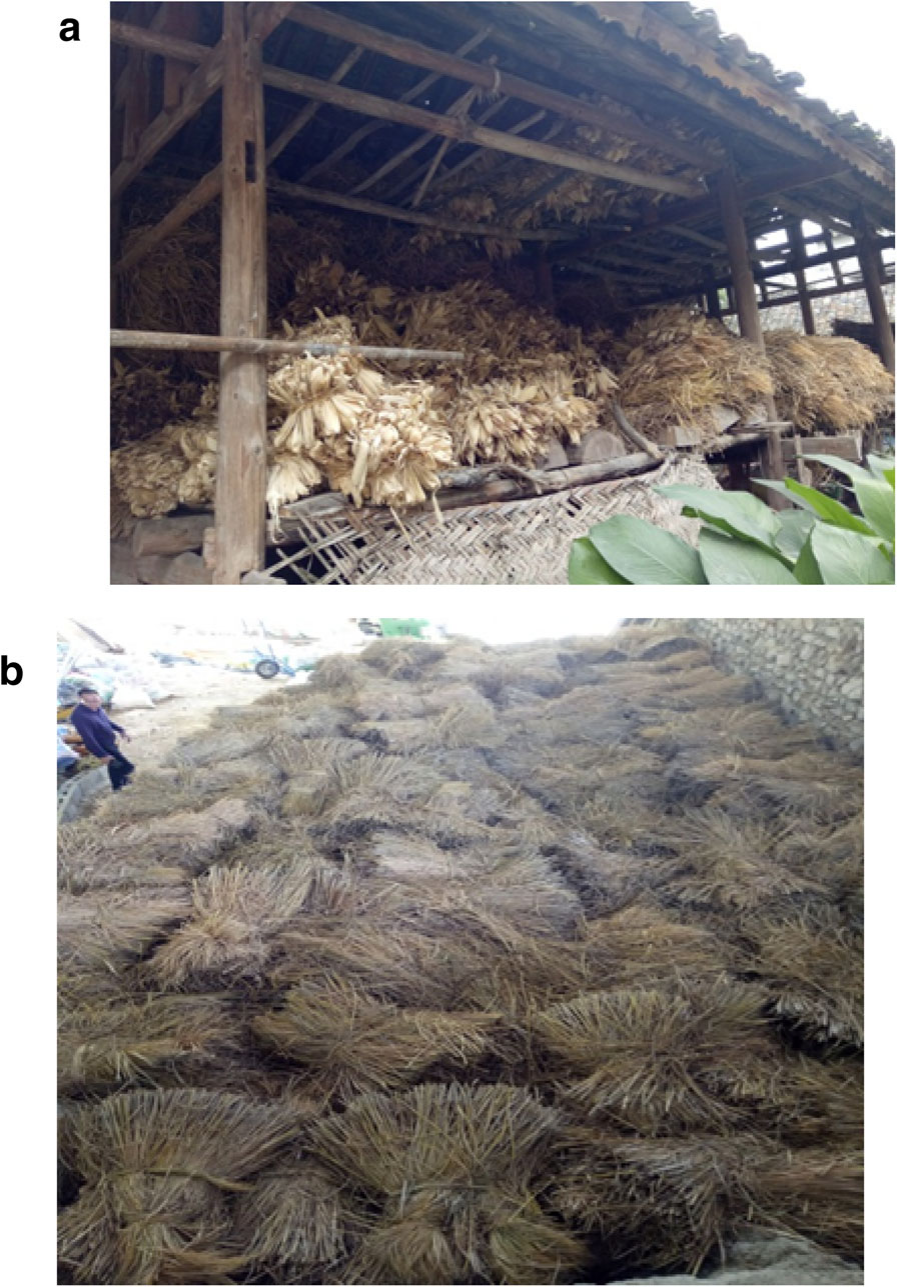
Local Buyi people saved dry fodders for domestic animals in winter. **a** Local Buyi people saved dry fodders for domestic animals in A’neng Village. **b** Honglong breeding farm saved dry fodders for domestic animals in Anlong County

Crop production and livestock are the major sources of cash income. There is a variety of livestock types and species in the Buyi villages, including cattle, pig, goat/sheep, chicken, horse, duck, goose, and mule. Figures 2 and 3 show images of the Buyi people’s domestic animals and the process of saving the dry crop fodders for livestock in winter.

Some of these were introduced from other regions by the prefectural Animal Husbandry Bureau and private sectors, such as pigs and cattle from cooperative breeding farms. But most were local breeds. Livestock is reared in cooperative farms for supermarkets and restaurants, while the residents generally rear livestock for their own consumption. Most local breeds have low yield, but good taste and adaptability.

### Data collection and analysis

**Fig. 4:**
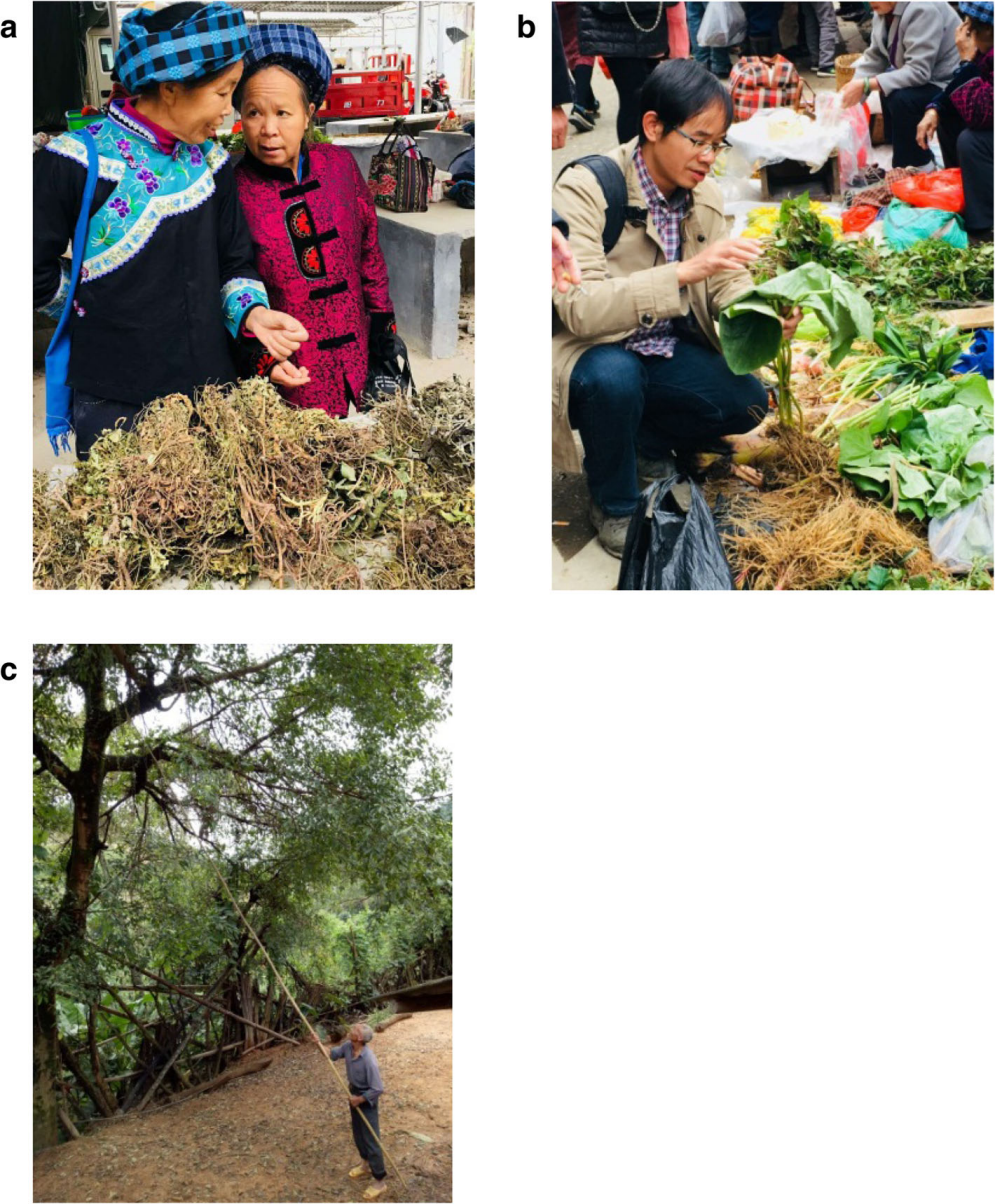
Local herbal medicinal markets (**a**, **b**) and medicinal plant collecting in a village (**c**). **a** Local Buyi Women are selling medicinal plants in Ceheng County herbal medicinal market. **b** One of the authors gathering information about plant use from Local herbal medicinal markets. **c** A local healer collecting medicinal plants in his home garden

The investigations of traditional knowledge of livestock illnesses and ethnoveterinary practices for the treatment of livestock diseases were conducted in 19 Buyi villages of Qianxinan Prefecture. The study area covered three counties: Anlong, Cehong, and Wangmo counties. The methods included direct observations, semi-structured interviews, key informant interviews, and focal group discussions (Fig. 4) [20, 21]. Figure 4 shows images of a local herbal medicinal market and medicinal plants collected by a local villager.

A total of 83 native Buyi people, 67 were male and 19 were female, including 44 people over 50 years of age, were interviewed (Table 2). All of informants are healers or veterinarians, farmers who have experienced in raising and managing livestock in our survey. Ethnobotanical investigations were conducted in 18 Buyi villages from 2017 to 2018. The primary content of the interviews consisted of “5W + H” questions (i.e., questions concerning what, when, where, who/whom, why, and how the subjects utilized veterinary medicinal plants) [21]. Each participant was acquired by presenting the main theme of the study to them, in order to gain their consent and trust, which allowed the participant to communicate more freely and openly. The recorded information was once again redisplayed to the informants to avoid errors and falsifications [22].

**Table 2:** Indicating the number and details of informants

Category	Total	%
Gender		
Male	67	77.9
Female	19	22.1
Age groups		
28~40	17	19.8
41~50	25	29.1
≥ 50	44	51.1
Occupation		
Farmers	46	53.5
Healer	19	22.1
Veterinarian	15	17.4
Other	6	7.0

Voucher specimens of the ethnoveterinary medicinal plants were collected by the first author, and identified by Dr. Chunlin Long, Dr. Bo Liu, and Dr. Qingsong Yang. The botanical names and their family names were corrected and verified through *The Plant List* website (http://www.theplantlist.org/) [23]. After collection, the plant specimens were treated and dried in the shade and were then mounted properly on the herbarium sheets for future reference. All voucher specimens were deposited in the Key Laboratory of Chemistry in Ethnic Medicinal Resources, State Ethnic Affairs Commission & Ministry of Education, School of Ethnomedicine & Ethnopharmacy, Yunnan Minzu University, Kunming, China.

For each of species collected, use reports (URs) (citations) were counted. The UR may be defined as the utilization of a part of a plant species for a disease, as mentioned by an informant [24]. To determine the informant consensus factor (Fic), the reported species were arranged in various groups, according to the ailment treated [25], and nine ailment categories were prepared from the data. The Fic was calculated as follows:

1 $$ \mathrm{Fic}=\left(\ \mathrm{Nur}-\mathrm{Nt}\right)/\left(\mathrm{Nur}-1\right) $$

where Nur indicates the number of citations in each use category and Nt represents the number of species cited.

## Results

### Respondents’ biographic details

The respondent age range was from 28 to 79 years old, with an average age of 51.3. Those who were older had more experience and traditional knowledge of animal production (Table 2). The education level of respondents was poor overall (most of them never went to middle school).

The younger respondents had higher education levels than the elders, and the females had lower education levels than the males. Those over 50 years of age were interviewed through the collection and investigation of the traditional knowledge of the veterinary medicines in the Buyi villages. Most of the people were farmers, healers, public officials, drivers, or pursuing small-scale businesses and jobs inside and outside the county.

Respondents aged between 40 and 50 were influenced by other modern veterinary medicinal knowledge. Some of them were trained by the government to use traditional veterinary medicine and western medicine to treat livestock. However, there are not many villagers who have the knowledge of traditional veterinary medicine between the ages of 20 and 40. Many young people go out for education or work and have no chance or time to study knowledge of traditional veterinary medicine.

### Veterinary disease category

In the study area, a total of 40 plant species were reported for use with gastrointestinal problems, with maximum use reports in 257 veterinary plant species (Table 3). Gastrointestinal problems were thus regarded as the most common disease category in the domestic animals, represented by abdominal pain, diarrhea, ruminal impaction, and digestive problems.

**Table 3:** Categories of informant consensus factors (FIC)

Medical categories	Number of species	Citations	FIC
Gastrointestinal disorders	40	257	0.85
Wound	26	197	0.87
Respiratory disorders	22	244	0.91
Miscellaneous	15	88	0.83
Parasitic diseases	10	43	0.78
Urinary	9	47	0.83
Tonic	8	57	0.88
Infectious diseases	7	56	0.89
Reproductive diseases	5	30	0.86

The main diseases of domestic animals in the study area include gastrointestinal diseases, infectious diseases, parasitic diseases, respiratory diseases, trauma and fracture, urinary, and other diseases. The sanitary conditions of local livestock houses are not very good. Meanwhile, the livestock mainly adopts the extensive breeding mode. The livestock eat unclean food and some poisonous plants, drink unclean water in field, so it cause discomfort or poisoning, and thus easily get gastrointestinal and parasitic diseases. Cattle, goat, and other domestic animals are primarily raised free range, not in captivity. There are abundant vegetation and bushes in local mountains and complex mountainous, which are prone to infectious diseases caused by traumatic wounds. At the same time, the climate during summer is hot and humid, so the trauma to the livestock makes them prone to infections by insects, flies, and other parasites.

The medical conditions that were not fully described by the interviewees were placed under the miscellaneous category. These include hepatitis, weakness, and hypotensive and abnormal conditions related to various organ systems of the animal bodies.

Sharma et al. [26] declared that if the Fic value is 1, then the local population exchanges views, ideas, and information about traditional medications, while if the Fic value is 0, then the opposite is true. The highest Fic values were recorded for respiratory disorders (0.91) followed by infectious diseases (0.89) and tonics (0.88) (Table 3).

The Fic value is an indicator showing the consent of the local people on a specific plant species and the efficacy of a certain taxa [27]. Table 3 shows that gastrointestinal diseases, trauma/fracture, and respiratory diseases are the most common diseases. Animal trauma and fracture diseases occur frequently in local livestock due to the mountainous terrain in the Buyi areas. Local veterinarians treat minor trauma infections and fractures in a timely manner.

 In particular, during summer rains, some livestock often fall into the valley and become injured; severe conditions can also lead to the death of livestock foraging in fields. The Buyi people usually use fresh medicinal plants (*Psammosilene tunicoides*, *Tinospora sinensi*, *Curcuma aromatica*, and *Ampelopsis delavayana*) to treat livestock for trauma and fracture. They grow these herbs in their courtyards which are easily accessible when they need to use them.

### Plant species for livestock illnesses

In our study, 122 plant species belonging to 60 families and 114 genera were documented. Table 4 presented details of the documented medicinal plants, including their botanical names, vernacular names, family names, specimen numbers, parts used, medicinal uses, and use reports (Table 4). The Compositae (Asteraceae, 11 species) family has the highest number of individual species that are used in ethnoveterinary practices, followed by Zingiberaceae (8 species) and Rosaceae (7 species).

**Table 4:** Inventory of ethnoveterinary plants in Qianxinan Prefecture

Voucher number	Scientific name	Vernacular name	Family name	Habit	Use part	Medicinal value	URs
Mxy01	*Achyranthes aspera* L.	Ye niuxi	Amaranthaceae	Herb	Whole	A whole plant is boiled and orally given to livestock for throat trouble.	10
Mxy02	*Aconitum vilmorinianum* Kom.	Xen caowu	Ranunculaceae	Shrub	Root	Dry roots are boiled and orally given to livestock for rheumatism.	5
Mxy03	*Adiantum capillus-veneris* L.	Tie xian pak	Pteridaceae	Fern	Whole	A whole plant is boiled and orally given to livestock for treatment of urethral disorder.	25
Mxy04	*Alpinia japonica* (Thunb.) Miq.	Ye yun	Zingiberaceae	Herb	Root	Roots are boiled and orally given to livestock for gastropathy.	22
Mxy05	*Ampelopsis delavayana* Planch. ex Franch.	Ye pu tao	Vitaceae	Climber	Root	Along with *Argyreia acuta* and *Coptis chinensis*, fresh leaves are boiled and orally given to livestock for prevention the foot-and-mouth disease virus.	24
Mxy06	*Anredera cordifolia* (Ten.) Steenis	Kau sanqi	Basellaceae	Climber	Stem	Fresh leaves are taken and crushed and made rake to cattle for treatment of fracture.	26
Mxy07	*Aralia spinifolia* Merr.	Fai ko yin	Araliaceae	Shrub	Root	Roots are boiled and orally given to livestock for treatment of fracture.	3
Mxy08	*Argyreia acuta* Lour.	Kau mo	Convolvulaceae	Climber	Leaf	Along with *Ampelopsis delavayana* and *Coptis chinensis*, fresh leaves are boiled and orally given to livestock for prevention the foot-and-mouth disease virus.	5
Mxy09	*Artemisia argyi* H.Lév. & Vaniot	Na ai	Compositae	Herb	Leaf	Fresh leaves are taken and crushed, and after that, are combined with fresh ginger and rubbed on cattle’s external tongue for oral disease and as anthelmintic.	48
Mxy10	*Asparagus cochinchinensis* (Lour.) Merr.	Kwe za zang	Asparagaceae	Herb	Root	Root of the herb is boiled and orally given to livestock for prevention respiratory ailments.	18
Mxy11	*Benincasa hispida* (Thunb.) Cogn.	Lwk fak	Cucurbitaceae	Climber	Park	Peel is boiled and orally given to livestock for treatment of hydropsy.	45
Mxy12	*Blumea balsamifera* (L.) DC.	Na nian	Compositae	Shrub	Leaf	Fresh leaves are taken and crushed, and after that, are combined with fresh ginger and *Artemisia argyi* and rubbed on cattle’s external tongue for oral disease.	38
Mxy13	*Centella asiatica* (L.) Urb.	Pak tin ma yong	Apiaceae	Herb	Whole	The whole plant is boiled and orally given to livestock for treatment of fractures.	22
Mxy14	*Curcuma longa* L.	Na yun yai	Zingiberaceae	Herb	Root	Fresh root of the herb is crushed and made to paste and coated afflicted part. This is also used to treat genital infection and problems.	36
Mxy15	*Curcuma phaeocaulis* Valeton	Ying jie	Zingiberaceae	Herb	Root	Fresh root of the herb is crushed and made to paste and coated afflicted part.	28
Mxy16	*Cymbopogon citratus* (DC.) Stapf	Na nang niu	Poaceae	Herb	Leaf	Along with *Polygonum perfoliatum*, a whole plant boiled and orally given to livestock for treatment of urinary trouble.	12
Mxy17	*Datura metel* L.	Man to lo	Solanaceae	Shrub	Fruit	Fresh fruits of the herb are crushed and mixed with fodder for the expulsion of intestinal worms and treated gastric problems	5
Mxy18	*Dischidia chinensis* Champ. ex Benth.	Gau nai fang	Apocynaceae	Shrub	Whole	A whole plant is boiled and orally given to cattle for treatment of pneumonia.	12
Mxy19	*Elephantopus scaber* L.	Na ditan	Compositae	Herb	Whole	A whole plant is boiled and orally given to livestock for treatment of cough and heat-clearing and detoxifying.	10
Mxy20	Eleutherine bulbosa (Mill.) Urb	Bu ling	Iridaceae	Herb	Tuber	Root of the herb is crushed and applied to livestock for treatment of wound.	3
Mxy21	*Kadsura longipedunculata* Finet & Gagnep.	Net tae	Schisandraceae	Climber	Root	Decoction is made from its fruits and orally administered to cattle for the treatment of cough.	8
Mxy22	*Leonurus japonicus* Houtt.	Yi mu cao	Lamiaceae	Herb	Whole	A whole plant is boiled and orally given to livestock for treatment of retained placenta.	48
Mxy23	*Lespedeza juncea var. sericea (Thunb.) Lace & Hauech*	tie zhaocou	Leguminosae	Shrub	Whole	Decoction is made from it stems and given orally to livestock for treatment of lung infections.	18
Mxy24	*Ligusticum sinense cv. Chuanxiong*	Chuan xiong	Apiaceae	Herb	Whole	Along with *Leonurus japonicus*, *Angelica sinensis*, and *Codonopsis pilosula*, dry roots are boiled and orally given to livestock for the retained placental removal.	24
Mxy25	*Lobelia seguinii* H.Lév. & Vaniot	Ye yan	Campanulaceae	Herb	Whole	Along with fresh leaves from *Solanum spirale* and fresh leaves are boiled and orally given to livestock for treatment of pulmonary infection.	5
Mxy26	*Lysionotus pauciflorus* Maxim.	Na ring tuo	Gesneriaceae	Herb	Stem	Decoction is made from it aerial parts and rally given to livestock for treatment of pulmonary infection.	6
Mxy27	*Ophioglossum vulgatum* L.	Na .lin ma	Ophioglossaceae	Herb	Whole	Root of the herb is crushed and applied to livestock for wound.	6
Mxy28	*Paris polyphylla var. chinensis*	Na chonglou	Melanthiaceae	Herb	Tuber	Decoction is made from its roots, and then, it is combined with fodder, given orally to livestock for heat-clearing and detoxifying.	36
Mxy29	*Perilla frutescens* (L.) Britton	Gau ao su	Lamiaceae	Herb	Whole	Dry leaves of herb is chopped, then boiled and orally given to livestock for treatment of gasteremphraxis.	40
Mxy30	*Plantago depressa* Willd.	Che qiancao	Plantaginaceae	Herb	Whole	A whole plant is boiled and orally given to livestock for treatment cough and heat-clearing and detoxifying.	53
Mxy31	*Polygonum perfoliatum* L.	Pak pag w	Polygonaceae	Herb	Whole	Along with *cymbopogon citratus*, a whole plant boiled and orally given to livestock for treatment of urinary trouble.	9
Mxy32	*Potamogeton distinctus* A.Benn.	Pjak na	Potamogetonaceae	Herb	Whole	Fresh leaves are boiled and orally given to livestock for treatment of pulmonary infection.	6
Mxy33	*Psammosilene tunicoides *W.C.Wu & C.Y.Wu	Ai tuo tuo	Caryophyllaceae	Herb	Whole	Root of the herb is crushed and applied to livestock for treatment of fracture.	26
Mxy34	*Pyrola calliantha* Andres	Lu ti cao	Ericaceae	Herb	Root	A whole plant is crushed and applied to livestock for treatment of fracture.	11
Mxy35	*Rhaphidophora decursiva* (Roxb.) Schott	Gau luo	Araceae	Shrub	Root	Fresh stem of the herb is crushed and mixed with fodder, then orally given to livestock as anthelmintic.	10
Mxy36	*Rotala rotundifolia* (Buch.-Ham. ex Roxb.) Koehne	Pajk na pie	Lythraceae	Herb	Whole	A whole plant is boiled and orally given to livestock for treatment of pulmonary infection.	8
Mxy37	*Schefflera elliptica* (Blume) Harms	Kau pie chai	Araliaceae	Shrub	Whole	Fresh leaves of the herb is crushed and applied to livestock for treatment of fracture.	5
Mxy38	*Sechium edule* (Jacq.) Sw.	kau yang gua	Cucurbitaceae	Climber	Leaf	Fresh vine of the herb is chipped dicing and mixed fodder, then orally given to livestock for promote digestion.	9
Mxy39	*Solanum spirale* Roxb.	Ma mao	Solanaceae	Shrub	Whole	Plant leaves and seeds are subjected to decoction and used topically for skin problems.	15
Mxy40	*Acmella paniculata* (Wall. ex DC.) R.K.Jansen	Ma tuo xue	Compositae	Herb	Leaf	A whole plant is boiled and orally given to livestock for treatment of toothache.	13
Mxy41	*Stephania epigaea* H.S. Lo	Na teng	Menispermaceae	Climber	Root	Power dry bulb of the herb is boiled and orally given for the treatment of stomach trouble.	8
Mxy42	*Swertia diluta* (Turcz.) Benth. & Hook. f.	Zhang ya cai	Gentianaceae	Herb	Whole	A whole plant is boiled and orally given to livestock for treatment of hepatitis.	34
Mxy43	*Taxillus chinensis* (DC.) Danser	Fai jia	Loranthaceae	Climber	Leaf	Plant stems are subjected to decoction and used topically used for general body improvement.	28
Mxy44	*Tinospora sinensisi* (A.Rich.) Miers	Kau ling	Menispermaceae	Shrub	Stem	Fresh leaves of the herb is crushed and applied to livestock for treatment of fracture.	19
Mxy45	*Uncaria rhynchophylla* (Miq.) Miq. ex Havil.	Kau wo	Rubiaceae	Climber	Root	Fresh stem are taken and decoction is made and orally given to livestock for abirritation.	20
Mxy46	*Verbena officinalis* L.	Pak yau piu	Verbenaceae	Herb	Whole	A whole plant is boiled and orally given to livestock for prevention of common cold.	43
Mxy47	*Xanthium strumarium* subsp. *sibiricum* (Patrin ex Widder) Greuter	Na fie fa	Compositae	Herb	Seed	Along with sulfur, dry fruits are powered and coated for treatment of fungal infection.	41
Mxy48	*Zingiber officinale* Roscoe	Yun	Zingiberaceae	Herb	Root	Bulb is crushed and mixed with fodder and orally given to livestock for promotion stomach digestion and against digestive disorders, for flatulence, and as appetizer.	56
Mxy49	*Sophora tonkinensis* Gagnep.	Shan dou gen	Leguminosae	Shrub	Root	Decoction is made from its roots, and orally given to livestock for treatment of respiratory infection.	21
Mxy50	*Plantago asiatica* L.	Na cheqian	Plantaginaceae	Herb	Whole	A whole plant is boiled and orally given to livestock for treatment of pulmonary infection.	53
Mxy51	*Berberis diaphana* Maxim.	San kezhen	Berberidaceae	Shrub	Root	Decoction is made from its roots, and orally given to livestock for treatment of diarrhea.	31
Mxy52	*Mahonia fortunei* (Lindl.) Fedde	Vai tang	Berberidaceae	Shrub	Root	Decoction is made from its roots, and orally given to livestock for treatment of gastric and throat troubles.	21
Mxy53	*Lonicera japonica* Thunb.	Kau zet ma	Caprifoliaceae	Climber	Flowers	Decoction is made from its roots, and orally given to livestock for treatment of throat troubles.	49
Mxy54	*Taraxacum mongolicum* Hand.-Mazz.	Na binba	Compositae	Herb	Whole	Decoction is made from its roots, and orally given to livestock for treatment of respiratory troubles.	52
Mxy55	*Coptis chinensis* Franch.	Na xam xen	Ranunculaceae	Herb	Root	Along with *Ampelopsis delavayana* and *Argyreia acuta*, fresh roots are boiled and orally given to livestock for prevention the foot-and-mouth disease virus.	46
Mxy56	*Senecio scandens* Buch.-Ham. ex D.Don	Na taxen	Compositae	Herb	Leaf	Fresh leaves are taken and decoction is made, and after that, the decoction is combined with *Lonicera japonica* and is used for livestock cold.	34
Mxy57	*Paris polyphylla* Sm.	Kue ta ma	Melanthiaceae	Herb	Root	Decoction is made from its roots, and then rubbed on animal’s external tongue for prevention of foot-and-mouth disease virus.	38
Mxy58	*Sanguisorba officinalis L.*	Na tuo ling	Rosaceae	Herb	Root	Dry roots are boiled and orally given to cattle for treatment of diarrhea, along with dry root of *Bletilla striata* and *Mahonia fortunei.*	5
Mxy59	*Bletilla striata* (Thunb.) Rchb.f.	Na to xen	Orchidaceae	Herb	Whole	A whole plant is boiled and orally given to livestock for treatment of diarrhea.	3
Mxy60	*Sophora flavescens* Aiton	ku tsm	Leguminosae	Herb	Root	Decoction is made from its roots and orally given to livestock for treatment of diarrhea.	36
Mxy61	*Aconitum carmichaelii* Debeaux	Za tau nu	Ranunculaceae	Herb	Root	Decoction is made from its rhizomes, and orally given to livestock for curing gastric distension.	5
Mxy62	*Notopterygium incisum* K.C.Ting ex H.T.Chang	Na tau	Apiaceae	Herb	Root	Fresh root of the herb is crushed and made to paste and coated to afflicted part.	3
Mxy63	*Heracleum hemsleyanum* Diels	Na tau long	Apiaceae	Herb	Root	Fresh root of the herb is crushed and mixed with made to paste and coated to afflicted part.	12
Mxy64	*Andrographis paniculata* (Burm.f.) Nees	Na ao ten	Acanthaceae	Herb	Whole	Decoction is made from its roots and orally given to livestock for treatment of inflammation.	37
Mxy65	*Scutellaria baicalensis* Georgi	Na lim	Labiatae	Herb	Root	Decoction is made from its roots and orally given to livestock for treatment of inflammation.	18
Mxy66	*Phellodendron amurense* Rupr.	Fai nang	Rutaceae	Tree	Bark	Decoction is made from its roots and orally given to livestock for treatment of inflammation.	23
Mxy67	*Citrus reticulata* Blanco	Ma kam	Rutaceae	Tree	Peel	Decoction is made from its peel fruit, and mixed peel fruit form *Citrus aurantium*, and after that orally given to livestock to help digestion.	12
Mxy68	*Citrus aurantium* L.	Ma puk	Rutaceae	Tree	Immature fruit	Decoction is made from its peel fruit, and mixed peel fruit form *Citrus reticulata*, and after that orally given to livestock to help digestion.	17
Mxy69	*Asarum heterotropoides* F.Schmidt	Pak lin kai	Aristolochiaceae	Herb	Whole	Powder of its roots is orally given for the treatment of cough.	15
Mxy70	*Hedychium coronarium* J.Koenig	Yun xau	Zingiberaceae	Herb	Root	Roots are crushed and given to livestock for fracture.	18
Mxy71	*Gentiana manshurica* Kitag.	Pak nai	Gentianaceae	Herb	Tuber	Decoction is made from the whole plant, and orally given to livestock for hepatitis.	29
Mxy72	*Dryopteris setosa* (Thunb.) Akas.	Pak mo	Dryopteridaceae	Herb	Tuber	A whole plant is boiled and orally given to livestock for treatment of liver parasite.	12
Mxy73	*Vigna radiata* R.Wilczek	Tua Lwk	Leguminosae	Herb	Seed	Grinds mung beans and mixes with vegetable oil, then orally given to pig for treatment of nitrite poisoning.	9
Mxy74	*Hordeum vulgare* L.	Xau mak	Gramineae	Herb	Malt	Decoction is made from its malt, and orally given to livestock for curing digestive complaints.	19
Mxy75	*Raphanus raphanistrum* subsp. *sativus* (L.) Domin	Lwk pok	Brassicaceae	Herb	Seed	Seed of the herb is fried and orally given to cattle for treatment of ruminal impaction.	36
Mxy76	*Cynanchum wilfordii* (Maxim.) Hemsl.	Za tau pin	Apocynaceae	Climber	Tuber	Fresh roots of the herb is crushed and orally given to cattle for treatment of ruminal impaction.	15
Mxy77	*Rheum palmatum* L.	Na ta baw	Polygonaceae	Herb	Root	Decoction is made from its rhizomes, and orally given to livestock for treatment of gastric distension.	10
Mxy78	*Acronychia acronychioides* (F.Muell.) T.G.Hartley	Fai ton ling	Rutaceae	Tree	Fruit	Decoction is made from its fruit, and orally given to livestock for curing gastric distension.	45
Mxy79	*Poria cocos*	Fu ling	polyporaceae	Herb	Fruit body	Decoction is made from its fruit, and orally given to livestock for invigorating stomach.	3
Mxy80	*Saussurea costus*(Falc.) Lipsch.	Mu xiang lao	Compositae	Herb	Root	Decoction is made from its roots, and orally given to livestock for curing digestive complaints.	14
Mxy81	*Alisma plantago-aquatica* L.	Fai bu	Alismataceae	Herb	Whole	Decoction is made from its bulb, and orally given to goat for diuretic swelling.	10
Mxy82	*Akebia quinata* (Houtt.) Decne.	Ta xan	Lardizabalaceae	Climber	Root	Powder of its stem is boiled and orally given to livestock for diureses and promoting lactation.	21
Mxy83	*Ligusticum tachiroei* (Franch. & Sav.) M. Hiroe & Constance	Pajk cong	Apiaceae	Herb	Root	Decoction is made from its bulb, and orally given to livestock for treatment of traumatic injury.	22
Mxy84	*Curcuma aromatica* Salisb.	Yujin	Zingiberaceae	Herb	Root	Roots are crushed and given to livestock for fracture.	19
Mxy85	*Alpinia officinarum* Hance	Ye yin ang	Zingiberaceae	Herb	Tuber	Roots are crushed and given to livestock for fracture.	15
Mxy86	*Curcuma kwangsiensis* S.G.Lee & C.F.Liang	Bae tse tse	Zingiberaceae	Herb	Tuber	Decoction is made from its roots, and orally given to livestock for treatment of gaseous distention.	27
Mxy87	*Eriobotrya japonica* (Thunb.) Lindl.	Ma pa	Rosaceae	Tree	Leaf	Fresh leaves of the herb is crushed and mixed with fodder and applied to livestock for treatment of lung complexities.	10
Mxy88	*Dipterocarpus turbinatus* C.F.Gaertn	Bin pian	Dipterocarpaceae	Tree	Borneol	Dry resin of herb is coated to livestock for exterior wound and sore throat.	3
Mxy89	*Codonopsis pilosula* (Franch.) Nannf.	Kau ling o	Campanulaceae	Herb	Root	Decoction is made from its roots, and orally given to livestock for general body improvement.	5
Mxy90	*Atractylodes macrocephala* Koidz.	Za fai xau	Compositae	Herb	Tuber	Powder of its roots is boiled and orally given for the treatment of stomach problems.	9
Mxy91	*Glycyrrhiza uralensis* Fisch.	Kan tshua	Leguminosae	Herb	Root, stem	Decoction is made from its roots, and orally given to livestock for treatment of digestive tract problem.	37
Mxy92	*Angelica sinensis* (Oliv.) Diels	Pak tam	Apiaceae	Herb	Root	Powder of its roots is orally given to animals, i.e., goat, sheep, and cow as tonic.	14
Mxy93	*Rehmannia glutinosa* (Gaertn.) DC.	Na tuo ran	Plantaginaceae	Herb	Root	Powder of its roots is boiled and orally given for treatment sore throat.	3
Mxy94	*Paeonia lactiflora* Pall.	Shao yao	Paeoniaceae	Herb	Root	Powder of its roots is boiled and orally given for treatment of stomachache. Powder is made and combined with flour which is used as tonic and for the treatment of cough in goat and cow.	3
Mxy95	*Punica granatum* L.	Ma li san	Lythraceae	Tree	Peel	Powder of its peel is boiled and orally given for treatment of stomachache and the removal worms.	19
Mxy96	*Areca catechu* L.	Ma sai kai	Arecaceae	Tree	Seed	Powder of its seed is orally given to livestock for the removal of worms and disperse accumulation.	5
Mxy97	*Quisqualis indica L.*	Shi jun zi	Combretaceae	Shrub	Fruit	Seed of the herb is fried and evigated, then mixed with fodder and orally given for the expulsion of intestinal roundworm.	6
Mxy98	*Allium sativum* L.	Lwk soi	Amaryllidaceae	Herb	Root	Powder of its roots is orally given to animals, i.e., goat, sheep, for treatment of infection. Bulb is crushed and mixed with way to administered orally in order to rate of fertility in domestic animals.	47
Mxy99	*Polygonatum sibiricum* F.Delaroche	Huang jing	Asparagaceae	Herb	Tuber	Along with fodder, root of the herb is crushed and applied to livestock for tonic.	35
Mxy100	*Crataegus pinnatifida* Bunge	Ma o	Rosaceae	Tree	Fruit	Decoction is made from its fruits, and orally given to cattle for treatment of ruminal impaction.	13
Mxy101	*Magnolia officinalis* Rehder & E.H.Wilson	Fai ton xen	Magnoliaceae	Tree	Bark	Decoction is made from its barks, and orally given to cattle for treatment of ruminal impaction.	5
Mxy102	*Veratrilla baillonii* Franch.	Na xen qiao	Gentianaceae	Herb	Root	Powder of its roots is orally given to foal for treatment diarrhoea.	8
Mxy103	*Dioscorea bulbifera* L.	Ye lwk jeu	Dioscoreaceae	Herb	Tuber	Fresh leaves of the herb is crushed and orally given to cattle for treatment of ruminal impaction.	4
Mxy104	*Litsea cubeba (*Lour.) Pers.	Ma ye gai	Lauraceae	Tree	Seed	Fruits of the herb are fried and evigated, then mixed with fodder and orally given for treatment of bilharziasis.	3
Mxy105	*Prunus persica* (L.) Batsch	Ma tau	Rosaceae	Tree	Leaf	Fresh leaves of the herb is crushed and orally given to cattle for treatment of ruminal impaction.	32
Mxy106	*Cannabis sativa* L.	Dai wai	Cannabaceae	Herb	Seed	Powder of its seed is orally given to livestock for embellish aperient bowel function flour. This remedy is given to domestic animals as refrigerant and also given to the cattle suffering for genital prolapsed.	3
Mxy107	*Glycine max (*L.) Merr.	Lwk tue	Leguminosae	Herb	Seed	Soybean milk is made from the herb and orally given to livestock for treatment of salt poisoning.	15
Mxy108	*Elsholtzia fruticosa* (D.Don) Rehder	Fai tuo rong	Lamiaceae	Herb	Flowers	Powder is made from flowers and applied topically to all domestic animals to treat dermal problems, as anti-inflammatory.	2
Mxy109	*Pachyrhizus erosus* (L.) Urb.	Di gua	Leguminosae	Herb	Stem	Decoction is made from it fresh stem and given orally to livestock for appetite.	6
Mxy110	*Geum aleppicum* Jacq.	Na no xen	Rosaceae	Herb	Whole	Powder is made from whole plant and applied topically to all domestic animals to treat dermal problems, as anti-inflammatory.	18
Mxy111	*Potentilla kleiniana* Wight & Arn.	Na to paejk	Rosaceae	Herb	Whole	Decoction is made from it fresh stem and given orally to cattle against cough, fever, and pain.	12
Mxy112	*Ardisia crispa* (Thunb.) A.DC.	Bai liangjin	Primulaceae	Herb	Root	Decoction is made from its roots, and orally given to livestock for treatment of diarrhea.	43
Mxy113	*Reynoutria multiflora* (Thunb.) Moldenke	Kau tan	Polygonaceae	Herb	Root	Along with bark of *Quercus acutissima*, decoction is made from its roots and given orally to cattle against diarrhea.	39
Mxy114	*Kalimeris indica* (L.) Sch.Bip.	Na tuo po	Compositae	Herb	Whole	Fresh leaves decoction is orally given for gastric troubles and as diuretic.	9
Mxy115	*Habenaria davidii* Franch.	Kwe dag kai	Orchidaceae	Herb	Tuber	Decoction is made from its roots, and orally given to cattle for treatment of urinary tract infections.	21
Mxy116	*Nicotiana tabacum* L.	Jion baw	Solanaceae	Herb	Leaf	To relieve the external parasite, the decoction of its leaves is used for animal bathing especially goat and cow. Decoction is made from its leaves, and it is applied externally on animal’s body and then rubbed for external parasites. Infusion of its leaves is drenched via nostrils against leech infestation in cows.	4
Mxy117	*Ainsliaea grossedentata* Franch.	Na pie can	Compositae	Herb	Whole	Fresh leaves of the herb is crushed and placed on wound for blood clotting.	1
Mxy118	*Talinum paniculatum (*Jacq.) Gaertn.	Ye zwn swn	Talinaceae	Herb	Root	Decoction is made from its roots and orally given to cattle for poisoned by *Cyclobalanopsis* green leaves.	31
Mxy119	*Schisandra propinqua* (Wall.) Baill.	Kau nang xeu	Schisandraceae	Shrub	Root	First make a paste of stem and external application to livestock for wound healing process.	21
Mxy120	*Quercus acutissima* Carruth.	Fai mai fen	Fagaceae	Tree	Root	Along with root of *Fallopia multiflora*, decoction of its bark is given orally daily to livestock for diarrhea.	10
Mxy121	*Libanotis seseloides* (Fisch. & C.A. Mey. ex Turcz.) Turcz.	Pjak an	Apiaceae	Herb	Whole	Decoction of its leaves is given orally to horse for the treatment of cough.	34
Mxy122	*Aesculus chinensis* Bunge	Ma ling xau	Sapindaceae	Tree	Seed	Decoction is made from its fruits and boiled, then orally given to livestock for treatment diarrhea.	16

Table 4 shows that 9 families have 2 species and 38 families have only 1 species respectively. The resources of the medicinal plants are abundant in the Buyi villages, and that local residents collect a diversity of medicinal species. The rationale for the high use of Compositae (Asteraceae) species in the current study, though based on traditional evidence, may be that Compositae has chemical constituents such as phenolics, poly phenolics, lectins, alkaloids, terpenoids, and flavonoids, which have antimicrobial potentials [28, 29]. As one of the largest families of seed plants over the world, the Compositae plants are easily available in local communities. The biomass and population sizes of Compositae plants are usually very large. Some species have multiple uses, for example, *Taraxacum mongolicum* is commonly used as wild edible herbs by local villagers. The villagers can also market some Compositae herbal products as a source of income, such as *Saussurea costus*, *Atractylodes macrocephala*, *Senecio scandens*, and *Kalimeris indica*.

### The life forms and used parts of plants for ethnoveterinary purposes

**Fig. 5:**
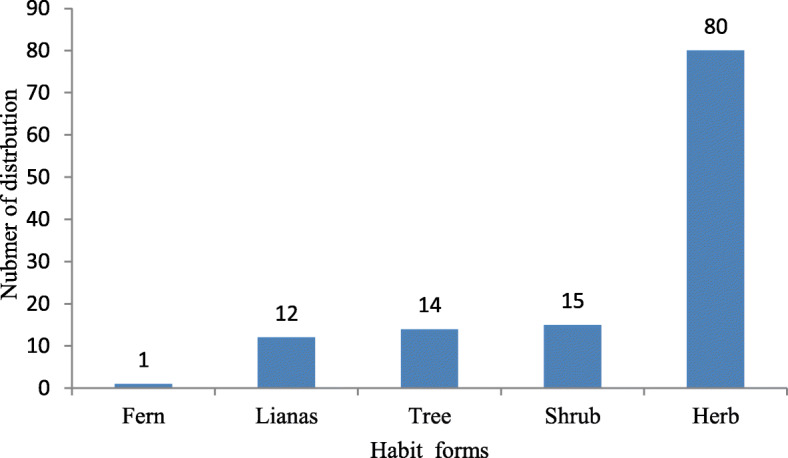
The number of distribution habit forms of ethnoveterinary plant species

Analysis of the growth forms of these medicinal plants revealed that the herbs constituted the largest number or proportion, with 80 species (65.6%), followed by 15 shrubs (12.3%), 14 trees (11.5%), 12 lianas (9.8%), and 1 fern (0.8%), as shown in Fig. 5.

**Fig. 6:**
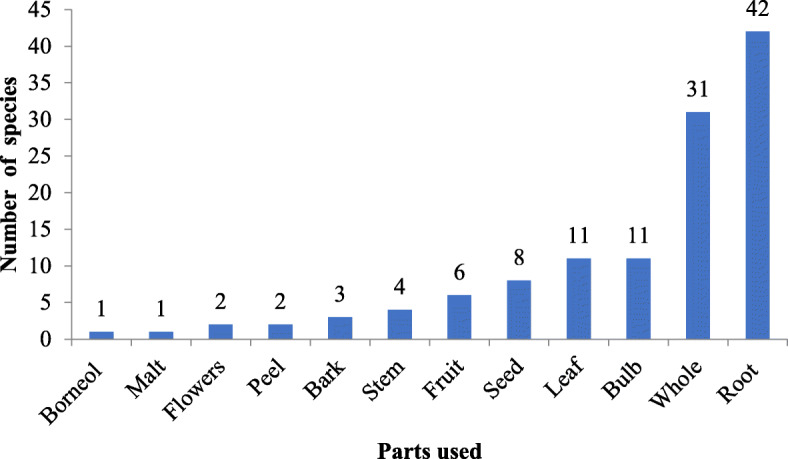
The used parts of ethnoveterinary plants in the Buyi areas

The roots were the plant parts most frequently used, constituting 34.4%, followed by whole plant (25.4%), bulb (9%), leaf (9%), and mixed plant parts (22.2%) (Fig. 6).

### The URs value of ethnoveterinary medicinal plants

Key informants declared extensive uses of the following: *Zingiber officinale* (56 URs), followed by *Plantago depressa* (54), *Plantago asiatica* (53), *Taraxacum mongolicum* (52), *Lonicera japonica* (49), *Artemisia argyi* (48), *Leonurus japonicas* (48), *Allium sativum* (47), *Coptis chinensis* (46), and *Benincasa hispida* (45) (Table 5). Medicinal plants with high URs strengthen the concept that such species are more significant to the local population and useful when sharing traditional knowledge. In this study, *Z*. *officinale* was found to be used to treat the common cold, abdominal pains, coughs, and vomiting in domestic animals. The usage mode of medicinal plant species by one community is different from that of other communities, due to the difference in traditional knowledge [30]. Previous literature has shown that gingerols of *Z*. *officinale* were used to antagonize the inflammatory effects of *Pinella pedatisecta* and *Arisaema heterophyllum* [31]. The decoction of *Z*. *officinale* tubers was also used for abdominal pain and to enhance body temperatures in the study area [32]. Similarly, the genus *Plantago*, which is known to have more than 200 species, is used extensively all over the world as a promising functional food resource, and for various remedies [33].

**Table 5:** Top 20 ethnoveterinary plant species with the highest use reports

Botanical name	Disease (or illness)	URs
*Zingiber officinale*	Digestive disorders, flatulence, fever	56
*Plantago depressa*	Fever	54
*Plantago asiatica*	Pulmonary infection	53
*Taraxacum mongolicum*	Respiratory problems	52
*Lonicera japonica*	Throat troubles	49
*Artemisia argyi*	Oral disease, anthelmintic	48
*Leonurus japonicus*	Postpartum extravasated blood	48
*Allium sativum*	Bacterial infection, fertility	47
*Coptis chinensis*	Fever	46
*Benincasa hispida*	Hydropsy	45
*Evodia rutaecarpa*	Gastric distension	45
*Verbena officinalis*	Common cold	43
*Ardisia crispa*	Diarrhea	43
*Xanthium sibiricum*	Fungal infection	41
*Perilla frutescens*	Gasteremphraxis	40
*Fallopia multiflora*	Diarrhea	39
*Blumea balsamifera*	Oral disease	38
*Paris polyphylla*	Fever	38
*Andrographis paniculata*	Fever	37
*Glycyrrhiza uralensis*	Digestive tract problem	37

In the present study, *Plantago depressa* and *P*. *asiatica* were used for various livestock problems. For instance, these plants were effective for digestion, diarrhea, and common colds, when mixed with *Berberis diaphana*, *Lonicera japonica*, and *Taraxacum mongolicum*. Pneumonia was also treated by providing their seeds to the animals, while it is also utilized to treat jaundice and as a diuretic [34, 35].

The flowers of *Lonicera japonica* are taken for the treatment of cough, fever, diarrhea, and swollen carbuncles [36]. Literature is scarce, regarding the use of *Lonicera japonica* as an anti-parasitic, and this highlights the unique use of this plant species in this study area. There is a familiarity among the local communities with similar uses. Decoction of the flowers of *Lonicera japonica* was used for cattle, goats, and sheep as an anti-parasitic, when mixed with *Asarum heteropoides*, *Alisma plantago*-*aquatica*, and peel pomegranate (*Punica granatum*). Published literature has indicated that the plant is also used for fever, cough, hepatoma cells, and mycoplasma gallisepticum infections [37, 38].

The fresh leaves of *Artemisia argyi* are crushed, and combined with fresh ginger and rubbed on the cattle’s tongue for oral diseases. The whole plant is used as an anthelmintic and anti-abortifacient for livestock. *Leonurus japonicas* is a common clinical medicine that activates blood circulation and regulating menstruation, inducing diuresis to alleviate edema, reducing fever, and detoxification. It has been used to treat menoxenia, dysmenorrhea, amenorrhea, lochia, edema of the body, oliguresis, sores, ulcerations, and other diseases in both humans and animals [39]. A whole plant of *Leonurus japonicas* is boiled and orally given to pigs to treat retained placenta.

The San-Huang mixture is composed of *Coptis chinensis* (Huang-lian), *Scutellaria baicalensis* (Huang-qin), and *Phellodendron amurense* (Huang-bai). It is widely used for the treatment of inflammation and fever of animals. Published literature has indicated that the San-Huang Xiexin decoction promotes good functional outcomes for acute ischemic strokes [40]. Several other plants were found to be popular as veterinary medicines in the area investigated, based on their high URs, including *Tetradium ruticarpum*, *Verbena officinalis*, *Ardisia crispa*, *Xanthium sibiricum*, *Perilla frutescens*, *Fallopia multiflora*, *Blumea balsamifera*, *Paris polyphylla*, *Andrographis paniculata*, and *Glycyrrhiza uralensis*. For example, *Tetradium ruticarpum* is the most common ethnoveterinary medicinal plant in Southwest China, and its fruits are used for the treatment of gastrointestinal disorders, such as gastric distension, diarrhea, and stomach overload [41]. Recently, literature has indicated that evodiamine and rutaecarpine from *Tetradium ruticarpum* could be used for the treatment of liver diseases [42].

*Glycyrrhiza uralensis* is an important traditional Chinese herbal medicine. It is named “Gancao,” and has been used to treat various diseases by local veterinarians, such as respiratory ailments, inflammatory disorders, heartburn, gastritis, liver problems, and skin diseases [43, 44]. Polysaccharides are one of the active components of *G*. *uralensis*. Many studies have indicated that the major bioactive ingredients of *G*. *uralensis* include flavonoids, polysaccharides, and triterpene saponins [45]. The herbal powder of *G*. *uralensis* might have great potential for the medical management of dairy cows with retained placentas [46].

## Discussion

### Distribution of veterinary plants and ethnoveterinary knowledge

Medicinal plants for ethnoveterinary purposes are mostly harvested from the field and some are planted in the gardens near houses. Very few however are purchased, but some are bought from local medicinal markets [47]. In recent years, due to the large number of migrant workers and the implementation of the conversion of farmland to forests, the rural ecological environment has been greatly improved. The outflow of labor has exacerbated the aging of rural populations, and currently, there are elderly villagers and school-age children living in the rural areas. Most of the previous paths and roads in the local mountains have been overgrown with weeds, and the number of medicinal herbs in the forest is decreasing due to the increase in heliophilous plants, which makes it very difficult for the Buyi people to find medicinal plants in their local fields. They can only rely on memory to find some larger liana and arbor medicinal materials, which are processed into dry products after preservation.

Although roots took a big proportion in medicinal plants for animals, the Buyi people have developed appropriate management ways to sustain medicinal resources. They collect mature medicinal plants and leave the small ones, if the roots of the herbs are not dug up. Consequently, local veterinarians often cultivate medicinal plants near their houses, especially some perennial herbs and shrubs, such as *Curcuma longa*, *Ligusticum tachiroei*, *Curcuma phaeocaulis*, *Toricellia tiliaefolia*, and *Dischidia chinensis*.

Ethnoveterinary practices and knowledge of the Buyi people are mainly derived from the daily practice of livestock production, while the traditional veterinary medicine knowledge is passed down from generations among the aborigines, mainly through oral imparts [48]. In Qianxinan Prefecture, almost every Buyi village and township have official veterinarians and traditional healers. Most local veterinarians have been influenced to some extent by outside knowledge, and some local veterinarians have been trained by government husbandry agencies. We also found that old villagers had some previous veterinarian transcripts written in Chinese during the survey. Most veterinarians are male, which may be because they spend more time involved with domestic animal production and management than women. In our study, we found that older villagers have more rich traditional knowledge and they are familiar with veterinary medications, diagnoses processes, and treatment methods than all others. But there were no significant differences between the young and middle-aged people.

### Choice of treatment provider and traditional treatment methods

With the Chinese government attaching great importance to rural livelihood issues, and the poverty alleviation program by 2020, the local government attaches great importance to the development and support of animal husbandry in ethnic areas. Each Buyi village has an agricultural technician (veterinarian), an agricultural extension station in the township, and an agricultural and rural bureau in the county agency. Every month, the township government gives cash supplements to old retired veterinarians in the Buyi village, and the local residents respect people who have the knowledge about herbal medicine. Indigenous communities play significant roles in reporting the traditional use of medicinal flora. The Buyi people first asked village official agricultural technicians for help to deal with their sick livestock when choosing treatment providers, because they trusted the local veterinarians’ therapeutic methods. Most veterinarians are knowledgeable in the treatment of livestock with combined ethnoveterinary and western methods. For serious infectious diseases, such as foot-and-mouth disease, swine fever, and chicken fever, the residents must report them to the local administrative departments.

To control these highly infectious diseases, infected livestock will be killed, burned, and buried. Disease not only results directly in economic losses to the livestock but also requires local residents to spend cash to recover livestock holdings, and sometimes villagers may even have to change their livelihood strategies. Local residents are aware that prevention rather than treatment is preferable for many livestock diseases.

In the present survey, we recorded many traditional treatment experiences in Buyi villages. The pig industry is an important pillar of local animal husbandry, and their main source of meat. Piglet postweaning diarrhea is the most common disease in pig production in Qianxinan area that results in significant financial losses to pig production. The main cause of this diarrhea is due to the accumulation of enterotoxic *Escherichia coli* with their dietary changes [49, 50]. If piglets already have diarrhea, the Buyi people would first use antibiotics (penicillin and streptomycin) to treat the animal disease, and then use traditional remedies for the recovery treatments, and prevention of recurrent diarrhea. A decoction is made from herb roots (*Berberis diaphana*, *Verbena officinalis*, and *Sophora flavescens*), and then given orally to the weaned piglets for the prevention of diarrhea.

Stress responses are the most common disease in livestock production activities [51]. The livestock production activities, such as purchasing new livestock, moving them into new houses, and changing them from free range to captive, may induce stress responses in livestock. Livestock illnesses, such as fevers and diarrhea, can occur if they do not adapt properly to their new environment. Prevention is the main method to treat stress responses in livestock. The Buyi people make decoctions from the roots of *Sophora tonkinensis* and *Mahonia fortunei*, which is then administered orally to prevent stress responses in livestock. Most of the medicinal plants for animals are used by Buyi people to prevent diarrhea and colds, and to improve the immunity of livestock.

The Buyi people usually use traditional methods in livestock for the treatment of common fractures and traumas, such as *Uncaria rhynchophyll*, *Tinospora sinensisi*, and *Ampelopsis delavayana*, which are mashed and wrapped around the wound. If a wound becomes infected, the Buyi people first use anti-inflammatory medicines (e.g., sulfonamides) to treat the infection [52], and then use veterinary plants (e.g., *Curcuma aromatica*, and *Paris polyphylla*) for further treatment of the wound.

### Pharmacological evidences

Drugs derived from plants or their extracts have certain therapeutic properties. To replace antibiotics with suitable therapeutic agents, plants can be utilized to play an important role in combating bacterial pathogens. Currently, antibiotic resistance is an emerging global concern, and a research hotspot for veterinary and human medications [53]. After a ban on the use of antibiotics in China, as growth promoters in farm animals, an interest in alternative products with antibacterial or anti-inflammatory properties has increased. Hence, it is necessary to search for new compounds to combat antibiotic-resistant bacteria.

*Artemisia argyi* is a region-specific plant from northern temperate zones, especially in Asia. Both in vitro and in vivo studies have revealed that *A*. *argyi* phytochemicals afford various health-promoting potentials, including antioxidant, anti-cancer, anti-inflammatory, immunomodulatory, neuroprotective, anticoagulant, and anti-osteoporotic activities, as well as antimicrobial and insecticidal properties [54]. The whole plant is used as an insecticide; branches of the herb are used as battens in livestock houses to repel parasites in survey area. The fresh leaves are taken and crushed, and after that, are combined with fresh ginger and *Blumea balsamifera* and rubbed on cattle’s external tongue for oral disease and as anthelmintic by the Buyi villagers. *Curcuma aromatica* contains curcumene, sesquidentenol, camphor, and camphene. It functions to clear up congestion and fever and detoxifies and relieves pain [55]. Jeon et al. [56] investigated the effects of *C*. *aromatica* water extracts (CAW) in the stomachs of rats with ethanol-induced gastritis. CAW increased the production of prostaglandin Esub2/sub. These findings suggest that CAW protects against ethanol-induced gastric mucosa injury by increasing the antioxidant status. Fresh roots are crushed and given to livestock for fracture in Buyi villages. *Zingiber officinale* has been widely used in traditional medicines since ancient times, and is also utilized as a dietary supplement. Extracts of *Zingiber officinale* including zingiberol, zingiberene, gingerol, and shogaol have a variety of health functions, such as warming the stomach and accelerating blood circulation. The pharmacological actions of ginger include antioxidant, anti-tumor, anti-apoptosis, anti-inflammation, anti-hyperglycemia, anti-cough, and anti-colds effects [57]. It has been found that extracts obtained in alcohol and water from *Zingiber officinale* had significant inhibitory effects on *Salmonella* and *Staphylococcus aureus*, and also antihypercholesterolemic activities as well as diuretic potential [58]. Local veterinarians use ginger in a variety of ways. Ginger is a common veterinary medicine used by local veterinarians to treat diseases such as oral diseases, digestive diseases, and colds in livestock. Tubers are crushed and mixed with fodder and orally given to livestock for promotion stomach digestion and against digestive disorders, for flatulence and as appetizer. *Taraxacum mongolicum* has been used in traditional Chinese medicine and dietary applications throughout the history of China. It has been reported that alcoholic and aqueous extracts of *Taraxacum mongolicum* have antibacterial activities and certain bactericidal effects on pneumococci, meningococcus, diphtheria bacillus, pseudomonas aeruginosa, dysentery bacillus, and typhoid bacillus [59]. Decoction is made from its roots, and orally given to livestock for treating respiratory troubles used by local Buyi people. *Sophora flavescens*, also known as Kushen in Chinese, has been an important species in Chinese medicine since the Qin and Han dynasties. The root of *Sophora flavescens* has a long history in the traditional medicines of many countries, including China, Japan, Korea, India, and some European countries [60]. In China, *Sophora flavescens* has been used extensively, mainly in combination with other medicinal plants, to treat fever, dysentery, hematochezia, jaundice, oliguria, vulvar swelling, asthma, eczema, inflammatory disorders, ulcers, and diseases associated with skin burns [61]. Its main medicinal components are matrine and oxymatrine in alkaloids, which have function to inhibit tumor cells and improve immunity [62]. *Sophora flavescens* is a very important herb for local veterinaries. Decoction is made from its roots and orally given to livestock for treating diarrhea along with *Ardisia crispa*, and treating respiratory disorders along with *Taraxacum mongolicum* and *Lonicera japonica*.

More than 100 herbal medicines have been found to be effective in killing and suppressing viruses and bacteria, such as *Houttuynia cordata*, *Strobilanthes cusia*, *Lonicera japonica*, *Andrographis paniculata*, *Coptis chinensis*, *Juniperus tibetica*, and *Scutellaria baicalensis* which are used in veterinary clinics by the Buyi people. Glycyrrhizin and glycyrrhetinic acid have significant neutralizing effects on the tetanus toxin. Honeysuckle and forsythia extracts can not only inhibit bacteria but also fight against the damage of *Escherichia coli* to body.

### Prospects and challenges to traditional ethnoveterinary knowledge

It has been reported that modern veterinary medicine may result in animal bacterial resistance, drug residues in animals used for food, and other safety issues [63]. With the extensive application of modern veterinary drugs, the problems of drug residues and drug resistance have become increasingly serious, and consequently more attention is being paid to traditional ethnic veterinary drugs, and this is expected to continue to increase in the future. Ethnoveterinary medicine has resource advantages, product advantages (in its side effect are small, there is no drug resistance, it is not easy to produce drug residues, and there is a natural versatility), the advantage of the prevention and control of animal diseases (with multiple components, multi-function, multiple targets, advocacy and symptoms, prevention and control of the whole system, and a series of dialectical therapies rather than just for pathogens). Due to the advantages of ethnoveterinary medicine, and to protect and utilize traditional knowledge, it will be necessary to carry out scientific research on ethnoveterinary medicines that have important scientific and practical values. According to the statistics of the world health organization, at least 80% of developing countries mainly rely on local traditional medical experience to prevent and treat various diseases in humans and animals. In some remote and poor areas, traditional veterinary medicines play an important role in replacing and supplementing western medicines. Traditional veterinary medicines also play an important role in livestock production and development and are an effective method for farmers to treat domestic animal diseases.

Ethnoveterinary medicines in China are different from modern medicine and modern veterinary medicine but could gradually be interpreted by modern science and technology. Chinese veterinarians have played a powerful role in ensuring the breeding and development of domestic animals for thousands of years. There are still many ethnic groups in China who live in poor areas, and do not have access to modern medical facilities, and consequently, they still rely on traditional herbs to prevent diseases in their livestock and poultry [64].

With the rapid development of socio-economy, science and technology, changes in the living environment, and influence from the mainstream culture, the protection of medicinal plants and related traditional knowledge is now crucial. If we do not take appropriate measures and techniques to protect the traditional Buyi veterinary knowledge, it will disappear with the death of the older generation. The traditional knowledge of Buyi veterinary medicine is facing extinction challenges.

## Conclusions

Buyi people in Qianxinan Buyi and Miao Autonomous Prefecture of Southwest Guizhou are dependent on medicinal plants for ethnoveterinary practices. Traditional knowledge of medicinal veterinary systems is restricted to the farmers, veterinarians, healers, and elder community members. Traditional knowledge on ethnoveterinary medicine is related to the local social-cultural characteristics of the Buyi people and plays a pivotal role in livestock production. This traditional knowledge is at risk of disappearing due to the increasing extension of western veterinary medicines, livelihood changes, and mainstream cultural influences.

Hence, this study is an attempt toward the preservation of traditional ethnoveterinary knowledge. There are several medicinal plants, which are being used in the traditional herbal system for veterinary disorders. Some of these important plants are *Sophora flavescens*, *Berberis diaphana*, *Sophora tonkinensis*, *Verbena officinalis*, *Mahonia fortunei*, *Ardisia crispa*, and *Aesculus chinensis*. The ethnoveterinary uses of *Talinum paniculatum*, *Quercus acutissima*, and *Aesculus chinensis* were newly recorded from the prefecture. Compositae is the largest plant family in use for various livestock ailments. Thorough phytochemical and pharmacological investigations are required, by isolating its active compounds and testing for in vitro or in vivo efficacy of the abovementioned plants against the targeted veterinary diseases. Furthermore, critical toxicological investigations are also required to ensure safe and secure use of the documented ethnomedicines. In order to share and further maintain this knowledge, it is necessary to share and disseminate ethnoveterinary knowledge locally, both within and between communities. In some cases, inexpensive allopathic treatments may exist where no effective ethnoveterinary treatments can be verified. Finally, it is important to make ethnoveterinary medicine and knowledge an integral part of modern animal health care in the Buyi areas.

## Data Availability

Not applicable.
